# Baltimore College of Dental Surgery

**Published:** 1841-12

**Authors:** 


### Miscellaneous Notices.

*Baltimore College of Dental Surgery.*—Pofessor H. Willis Baxley, M. D., by whom the chair of Special Anatomy and Physiology in this institution, was so ably filled during the first course of lectures, resigned his connection with it about three weeks previous to the commencement of the second, and W. R. Handy, M. D., adjunct professor of Anatomy and Physiology in the Washington University of Baltimore, was elected to the vacancy occasioned thereby. In retiring from the institution, Professor Baxley, takes with him our sincere regards. As a lecturer and teacher of anatomy he ranks among the very first in this country. His abilities, however, and high professional attainments are well known and too highly appreciated to require any commendation from us, and while it is a source of deep regret that his official connection with the college has been interrupted, we cannot withhold an expression of gratification that it has not proceeded from any want of interest or confidence in the success of our enterprise, but from the imperative demands upon his time resulting from arduous professional duties. But, while we thus regret the loss of Professor Baxley's valuable services, we are happy to announce that his place has been filled by one eminently qualified to discharge its duties.—*Balt. Ed.*

*Catalogues of the Members of the American Society of Dental Surgeons,* may be had, neatly printed, on paper or parchment, by application to either of the editors of this journal.

